# Self-Healable PEDOT:PSS-PVA Nanocomposite Hydrogel Strain Sensor for Human Motion Monitoring

**DOI:** 10.3390/nano13172465

**Published:** 2023-08-31

**Authors:** Jie Cao, Zhilin Zhang, Kaiyun Li, Cha Ma, Weiqiang Zhou, Tao Lin, Jingkun Xu, Ximei Liu

**Affiliations:** 1 Jiangxi Key Laboratory of Flexible Electronics, Flexible Electronics Innovation Institute, Jiangxi Science and Technology Normal University, Nanchang 330013, China; caojie9905@163.com (J.C.); zhangzhilin1117@163.com (Z.Z.);; 2 School of Chemistry and Chemical Engineering, Jiangxi Science and Technology Normal University, Nanchang 330013, China; 3 Key Laboratory of Organic Chemistry in Jiangxi Province, Institute of Organic Chemistry, Jiangxi Science and Technology Normal University, Nanchang 330013, China; macha001@126.com; 4 Department of Orthopedics, Qilu Hospital, Cheeloo College of Medicine, Shandong University, Qingdao 266035, China; 5 School of Chemistry and Molecular Engineering, Qingdao University of Science and Technology, Qingdao 266042, China

**Keywords:** conducting polymer hydrogels, PEDOT:PSS, self-healing, strain sensors, wearable electronics

## Abstract

Strain sensors based on conducting polymer hydrogels are considered highly promising candidates for wearable electronic devices. However, existing conducting polymer hydrogels are susceptible to aging, damage, and failure, which can greatly deteriorate the sensing performance of strain sensors based on these substances and the accuracy of data collection under large deformation. Developing conductive polymer hydrogels with concurrent high sensing performance and self-healing capability is a critical yet challenging task to improve the stability and lifetime of strain sensors. Herein, we design a self-healable conducting polymer hydrogel by compositing poly(3,4-ethylenedioxythiophene):polystyrene sulfonate (PEDOT:PSS) nanofibers and poly(vinyl alcohol) (PVA) via both physical and chemical crosslinking. This PEDOT:PSS-PVA nanocomposite hydrogel strain sensor displays an excellent strain monitoring range (>200%), low hysteresis (<1.6%), a high gauge factor (GF = 3.18), and outstanding self-healing efficiency (>83.5%). Electronic skins based on such hydrogel strain sensors can perform the accurate monitoring of various physiological signals, including swallowing, finger bending, and knee bending. This work presents a novel conducting polymer hydrogel strain sensor demonstrating both high sensing performance and self-healability, which can satisfy broad application scenarios, such as wearable electronics, health monitoring, etc.

## 1. Introduction

Strain sensors that can mimic the sensory functions of human skin via converting mechanical deformation to electrical signals have attracted considerable attention in various fields [1,2,3], including electronic skins [4], soft robotics [5,6,7], and wearable electronic devices [8,9]. Depending on various sensing principles, strain sensors can be categorized as resistive, triboelectric, capacitive, and piezoelectric-type strain sensors. Due to their high sensitivity, wide monitoring range, simple device structure, and facile fabrication process, resistive-type strain sensors have attracted more interest [10]. Stimulated by external stress, the variation of resistive-type strain sensors originates from ratios between resistance and current [11]. The variation in sensor resistance can be affected by the design of various sensing materials, as well as microstructures [1]. Carbon-based materials, metallic nanomaterials (nanowires and nanoparticles), and conductive hydrogels are regarded as the most common sensing materials for strain sensors, owing to their tunable electrical performance, outstanding mechanical property, and biocompatibility. Corresponding microstructures, including island bridge structures, pleated structures, flexural structures, and serpentine structures, can maintain the robustness of conductive paths under high levels of strain by diluting the applied strain into small distance changes in the conductive filler, obtaining a wide detection range [12]. However, existing resistive-type strain sensors are prone to mechanical damage, aging, and failure during external stimulation [10,13], such as large deformation and extreme conditions, immensely deteriorating the sensing performance of these strain sensors, the accuracy of data collection, and shortening their lifetime [14,15,16].

Withstanding repeated mechanical stress and maintaining their functionality are regarded as significant requirements for resistive-type strain sensors. Simultaneously endowing strain sensors with excellent mechanical performance and self-healing capabilities to recover or repair themselves after being subjected to mechanical damage or deformation is an effective approach. Self-healing properties can enhance the durability and longevity of strain sensors, allowing them to maintain their functionality and accuracy over an extended period [17,18]. As a result, such strain sensors can continue to provide reliable data even in harsh or challenging conditions, making them highly valuable in various applications, such as structural health monitoring, wearable devices, and biomedical systems. Therefore, innovative efforts have been devoted to the design of high-performance sensing materials with self-healability after undergoing large deformations to repair the sensing performance and prolong the service life of strain sensors [4,10,19].

Conducting polymer hydrogels are attractive candidate materials for strain sensors due to their tissue-like mechanical compliance [20,21], intrinsic ionic and electronic conductivity [22], and biocompatibility [23]. For example, with a poly(3,4-ethylenedioxythiophene):polystyrene sulfonate (PEDOT:PSS)-poly(vinyl alcohol) (PVA) hydrogel prepared via freeze-casting and salting-out, our group recently fabricated a PEDOT:PSS-PVA hydrogel strain sensor with long-term strain sensing robustness [24]. Gu et al. also designed PEDOT:PSS-PVA hydrogel strain sensors with high stretchability and ultralow hysteresis via freeze–thawing to monitor various physiological signals [17,18]. Although such strain sensors demonstrate high sensing performance and negligible hysteresis, the reported strain sensors typically suffer from irreversible mechanical damage during large deformation. Hence, rendering PEDOT:PSS-based hydrogels with both high sensing performance and favorable self-healability is a pivotal yet challenging task to enhance the stability and lifetime of strain sensors. 

It is well known that the self-healability of conducting polymer hydrogels stems from non-covalent physical interactions or the reconstruction of reversible dynamic covalent bonds [17,18]. Physical interactions such as hydrogen bonding, π–π stacking, electrostatic interaction, and metal coordination can endow conducting polymer hydrogels with a certain self-healability [14,25,26], yet the resultant hydrogels usually display poor mechanical performance, limiting their widespread application in various fields. Reversible chemical interactions (including imine bonding, borate bonding, and Diels–Alder reaction) can form multiple reversible dynamic interactions within the hydrogel networks, but such a method may severely reduce the sensing performance due to chain immobilization. In contrast, physical–chemical dual crosslinking has become a promising tactic for preparing self-healable conducting polymer hydrogels owing to the formation of multiple reversible dynamic interactions [14,25]. It is expected that PEDOT:PSS-based conducting polymer hydrogels prepared via physical–chemical dual crosslinking will simultaneously possess favorable mechanical properties, high sensing performance, and excellent self-healability, ensuring the rapid repair of the network structure and functions [5,27,28].

Herein, we design a self-healable PEDOT:PSS-PVA nanocomposite hydrogel via the physical–chemical dual crosslinking of PEDOT:PSS nanofibers and PVA. The resultant hydrogel displays a high monitoring range (>200% strain), a low Young’s modulus (~24 kPa), rapid resistance self-recovery (0.23 s), and outstanding self-repair efficiency (>83.5%). We further prove that PEDOT:PSS-PVA conducting polymer hydrogel strain sensors possess low hysteresis (<1.6%) and a high gauge factor (GF = 3.18). To leverage the improved sensing performance of such sensors, we demonstrate PEDOT:PSS-PVA strain sensors as wearable electronic skins, which can accurately monitor various physiological signals, including swallowing, finger bending, and knee bending. Benefiting from these merits, the PEDOT:PSS-PVA strain sensor integrated with both high sensing performance and self-healability provides a new horizon to construct a platform for next-generation strain sensors.

## 2. Materials and Methods

### 2.1. Materials

PH1000 (Clevios^TM^, Heraeus Electronic Materials, Sigma-Aldrich (Shanghai, China) Trading Co., Ltd.), PVA (Mw 146,000–186,000, Shanghai Aladdin Biochemical Technology Co., Ltd., Shanghai, China), sodium tetraborate (≥99%, Shanghai Aladdin Biochemical Technology Co., Ltd.), and dimethyl sulfoxide (DMSO, Sigma-Aldrich (Shanghai, China) Trading Co., Ltd.) were directly used without any purification.

### 2.2. Preparation of Self-Healable PEDOT:PSS-PVA Nanocomposite Hydrogels

Commercial PH1000 is a colloidal dispersion solution in which PEDOT:PSS grains with different practice sizes (30–50 nm) are stably dispersed in water. Owing to the existence of electrostatic forces, PEDOT:PSS grains display a core–shell structure that is composed of an insoluble PEDOT-enriched core and a water-soluble PSS shell. Furthermore, phase separation between hydrophobic PEDOT and hydrophilic PSS will induce the aggregation of PH1000 solution. To break up this core–shell structure and thus obtain homogeneous PEDOT:PSS solutions, the PH1000 solution underwent vigorous agitation for 6 h at room temperature and was meticulously filtered through a filter with a precise pore size of 0.45 µm, effectively removing any impurities or particulate matter. To endow PEDOT:PSS with more controllable and scalable nanostructures, the freezing and successive drying method in which the PH1000 solution was frozen and submerged in liquid nitrogen was employed [29]. Then, 0.764 g of PEDOT:PSS nanofibers was dissolved in 10 mL of a binary solvent that consisted of deionized water (DI) and DMSO (Water:DMSO = 85:15 *v*/*v*), and then was mixed with syringes to obtain a viscous solution. Then, 0.7 g of PVA powders was poured into 9.3 g of DI water and stirred at 90 °C to obtain a transparent solution. Briefly, 0.1 g of sodium tetraborate was added to 9.9 mL of DI water under stirring to acquire the solution. Subsequently, the PEDOT:PSS-PVA solution was prepared by vigorously mechanical mixing the PEDOT:PSS nanofiber solution and viscous PVA solution, and filtered with a syringe filter (45 μm), which guaranteed that PEDOT:PSS and PVA yielded a homogeneous solution. Then, the PEDOT:PSS-PVA solution was degassed via centrifuging at 8000 r.p.m. for 10 min to obtain 1, 3, 5, 10, and 15 wt.% homogeneous solutions (Table S1). Subsequently, the chemical-crosslinking method that selects borax as the chemical agent was adapted to achieve the fast gelation of the PEDOT:PSS-PVA nanocomposite hydrogel.

### 2.3. Characterizations

FTIR spectroscopy (Perkin-Elmer Spectrum TWO infrared spectrophotometer, Waltham, MA, USA) was performed to analyze the chemical structure of PEDOT:PSS, PVA and PEDOT:PSS-PVA.

### 2.4. Mechanical Properties

The mechanical properties of PEDOT:PSS-PVA nanocomposite hydrogels with rectangular shapes with sizes of 20 × 80 mm were tested using a mechanical testing machine (ZQ-990LB, Zhiqu Precision Instrument, Dongguan, China) with a 5 N load cell. The Young’s modulus was calculated from 5–10% of the strain on the strain–stress curve. 

### 2.5. Sensing Performance

The sensing performance of PEDOT:PSS-PVA nanocomposite hydrogels, including the relative change in electrical resistance and gauge factor, was recorded using an LCR meter (Tonghui, TH2829C, Wuxi, China). The corresponding gauge factor (GF) was calculated as follows: GF = (∆*R*/*R*_0_)/ε, where ∆*R* and *R*_0_ represent the change in resistance after applying a strain ε and the initial resistance, respectively. 

### 2.6. Self-Healing Properties 

Self-healable PEDOT:PSS-PVA nanocomposite hydrogels were stained with methyl blue and rhodamine B. At room temperature, two separate hydrogels were stretched after contact for 30 s without external stimulation. Subsequently, electrical and mechanical recovery properties were investigated.

### 2.7. Preparation of Strain Sensors 

Proper encapsulation and packaging are vitally important for strain sensors to maintain stable performance, which can ensure minimal noise and signal drift. In this work, we selected 3M very-high-bond (VHB) tape with outstandingly strong adhesion and corrosion resistance as the encapsulation layer and PEDOT:PSS-PVA nanocomposite hydrogel as the sensing layer to fabricate hydrogel strain sensors with sandwich structures (3 cm × 2 cm).

### 2.8. Participant Recruitments 

Testing of the PEDOT:PSS-PVA nanocomposite hydrogel on humans (authorized by the Qilu Hospital of Shandong University (Qingdao, China) Medical Ethics Committee (KYLL-KS-2023128) does not affect living human health. The throat, hands, arms, and knee that are displayed in experiments are those of Z.Z. (the co-first author), who agreed for these images to appear here.

## 3. Results and Discussion

### 3.1. Design Principle of Self-Healable PEDOT:PSS-PVA Nanocomposite Hydrogel Strain Sensors

**Figure 1:**
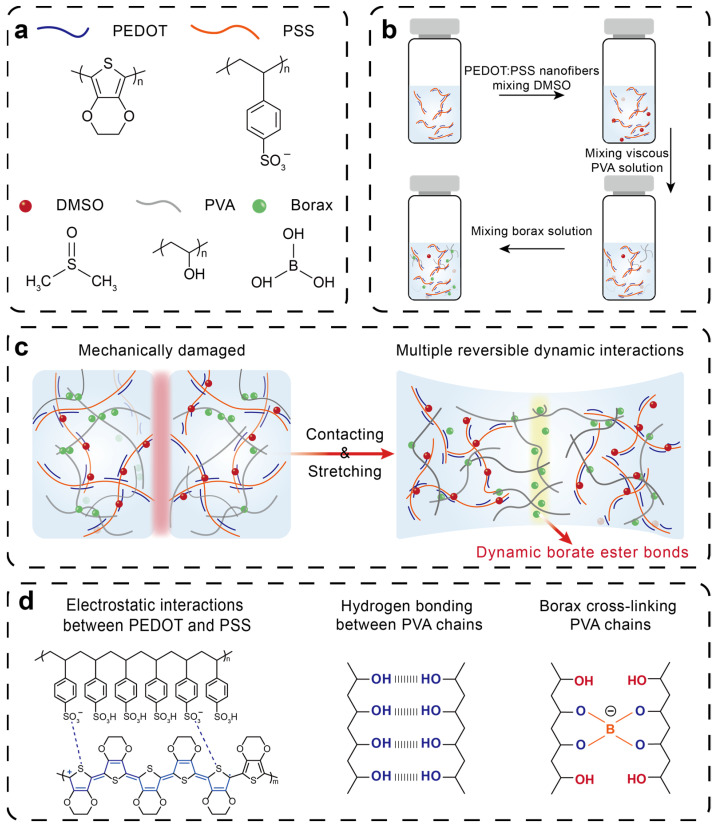
Schematic diagram of the formation mechanism and preparation of the self-healable PEDOT:PSS-PVA nanocomposite hydrogel. (**a**) Chemical structures of PEDOT, PSS, DMSO, PVA, and borax. (**b**) Schematic illustration of designing the PEDOT:PSS-PVA hydrogel. (**c**) The formation process of dynamic reversible borate ester bonds between PVA chains and borax. (**d**) The main multiple interactions within the PEDOT:PSS-PVA hydrogel.

Endowing strain sensors with both high sensing performance and self-healability is a considerable yet challenging task for improving the stability and lifetime of strain sensors. For a better design of strain sensors with high sensing performance and self-healability, we select PEDOT:PSS as the sensing material, and add a secondary dopant, dimethyl sulfoxide (DMSO), into PEDOT:PSS to improve the electrical performance of PEDOT:PSS, ensuring the production of strain sensors with favorable conducting pathways and a high signal-to-noise ratio. Motivated by physical–chemical dual crosslinking and multiple reversible dynamic interactions, we introduce PVA as the second polymer network into PEDOT:PSS nanofibers and select borax as the chemical crosslinking agent to prepare the PEDOT:PSS-PVA nanocomposite hydrogel with high sensing performance and self-healability (Figure 1a,b). The hydrophilic PSS and PVA matrix affords outstanding stretchability and excellent robustness to PEDOT:PSS-PVA nanocomposite hydrogels. This remarkable self-healability stems from three major molecular interactions including hydrogen bonding between PVA chains, electrostatic interactions between PEDOT and PSS, and dynamic borate ester bonding between borate and PVA chains (Figure 1c,d) [20,30,31]. As an increasingly significant strain is exerted, the distance between PEDOT:PSS nanofibers suffers from substantial expansion, destroying the continuity of conducting pathways. This disruption continues until the PEDOT:PSS-PVA nanocomposite hydrogel undergoes fracture. However, due to multiple reversible dynamic interactions, the fractured PEDOT:PSS-PVA nanocomposite hydrogel exhibits excellent self-healability, enabling the repair of the mechanical, electrical, and sensing properties of the PEDOT:PSS-PVA nanocomposite hydrogel. Additionally, the obtained PEDOT:PSS-PVA nanocomposite hydrogel is more susceptible to changes in strain, which can enhance the sensitivity and reduce the response time of strain sensors and further improve the sensing performance of strain sensors. Fourier transform infrared spectroscopy (FTIR) is carried out to explore the chemical structure of PEDOT:PSS-PVA nanocomposite hydrogels (Figure S1). All of these characteristic peaks of PEDOT:PSS and PVA can be observed in the PEDOT:PSS-PVA hydrogel with a significant peak shift. The characteristic peak at 3450 cm^−1^ in PVA is assigned to the stretching vibration of O−H groups [29]. Specifically, the PEDOT:PSS-PVA nanocomposite hydrogel displays a peak originating from C=O stretching vibration at 1660 cm^−1^, which is due to the existence of a number of boric ester bonds.

To harness the high sensing performance and self-healability of the PEDOT:PSS-PVA nanocomposite hydrogel, we further fabricate PEDOT:PSS-PVA hydrogel strain sensor-based wearable electronic skins to monitor various physiological signals, including swallowing, finger bending, and knee bending, which display repeatable and stable sensing performance. The practical application of PEDOT:PSS-PVA hydrogel strain sensors demonstrates the extensive potential applications of such hydrogels as next-generation strain sensors.

#### 3.1.1. Self-Healing Performance

To afford a more indicative view of the self-healing process of PEDOT:PSS-PVA nanocomposite hydrogel, varying samples including pure PEDOT:PSS hydrogel, PEDOT:PSS-PVA composite hydrogel, and PEDOT:PSS-PVA nanocomposite hydrogel are cut into two segments and the self-healing behavior is observed, with the aim of comparing their self-healability (Figure S2). After contact for 30 min, regular hydrogels like pure PEDOT:PSS and the PEDOT:PSS-PVA hydrogel do not exhibit obvious self-healing behavior (Figure S2a,b). In contrast, the PEDOT:PSS-PVA nanocomposite hydrogel in this work displays an effective self-healing ability and can withstand mechanical stretching after contact for only 30 s (Figure S2c). Physically crosslinked hydrogels are connected through weakened intermolecular forces that are the main cause of physically crosslinked hydrogels possessing poor self-healability, which are unable to provide sufficient energy to drive the self-repair process. The dynamic boronic-ester covalent bonds between PVA chains and borax [32,33,34], the hydrogen bonds between PVA chains, and the electrostatic interactions between PEDOT and PSS chains provide the self-healability to the PEDOT:PSS-PVA nanocomposite hydrogel, allowing such hydrogels to self-heal without external forces.

**Figure 2:**
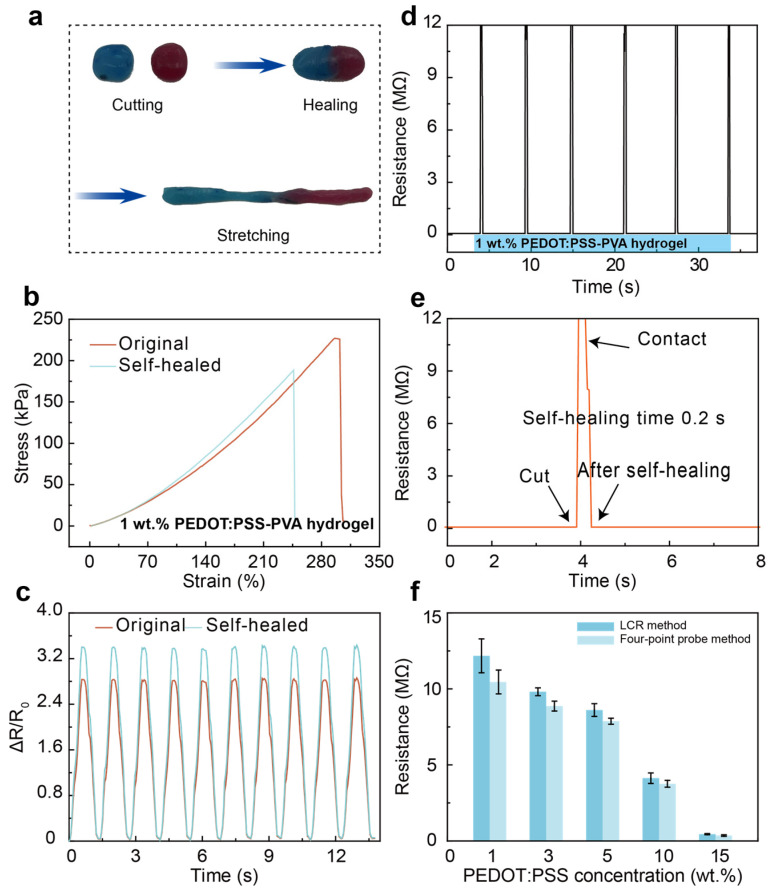
Self-healability of PEDOT:PSS-PVA nanocomposite hydrogels. (**a**) Macroscopically observed self-healing behavior of the PEDOT:PSS-PVA nanocomposite hydrogel by contacting and stretching. (**b**) Tensile stress–strain curves of the 1 wt.% PEDOT:PSS-PVA hydrogel before and after healing. (**c**) Relative resistance change of the 1 wt.% PEDOT:PSS-PVA hydrogel under the original and self-healed state. (**d**) Resistance recovery of the PEDOT:PSS hydrogel during the cutting-healing cycle and (**e**) the corresponding larger version. (**f**) Resistance measurement of PEDOT:PSS-PVA nanocomposite hydrogels with various PEDOT:PSS contents via LCR and the four-point probe method, respectively.

In addition, to quantitatively evaluate the self-healability of PEDOT:PSS-PVA nanocomposite hydrogels, the tensile stress–strain test is performed on the original and self-healed 1 wt. % PEDOT:PSS-PVA hydrogel and calculates the healing efficiency. The stress of the original PEDOT:PSS-PVA hydrogel is 298 kPa, and the stress of the self-healed hydrogel is 246 kPa for 30 s (Figure 2b), which demonstrates the outstanding self-healability of PEDOT:PSS-PVA hydrogels and displays favorable healing efficiency (>83.5%). Meanwhile, we examine the sensitivity of PEDOT:PSS-PVA hydrogel in the original and self-healing state (Figure 2c). The self-healable PEDOT:PSS-PVA exhibits a significant improvement in sensitivity. The slight enhancement in sensing performance after self-healing is due to the fact that the multiple reversible dynamic bonds are not fully repaired. Therefore, the PEDOT:PSS-PVA hydrogel undergoes greater resistance changes during stretching, resulting in an increment in sensitivity. The electrical performance of PEDOT:PSS-PVA hydrogel is afforded by the connection of intrinsically conducting PEDOT chains. When a piece of this hydrogel is cut off and then reconnected, the corresponding conducting polymer networks are connected and restore the electrical percolation network. As shown in Figure 2d,e and Figure S3, the PEDOT:PSS-PVA hydrogel exhibits a distinct difference in the electrical resistance under the severed and self-healing states. When the hydrogel is severed, the resistance instantly becomes infinitely large. Upon reconnection of the PEDOT:PSS-PVA hydrogel, its resistance can be restored to its initial value within 0.2 s. Resistance measurement of the PEDOT:PSS-PVA nanocomposite hydrogel with various PEDOT:PSS contents is explored by utilizing LCR and the four-point probe method, respectively (Figure 2f). Note here that the resistances of the PEDOT:PSS-PVA hydrogel with different methods are all within the same range.

#### 3.1.2. Mechanical Performance

**Figure 3:**
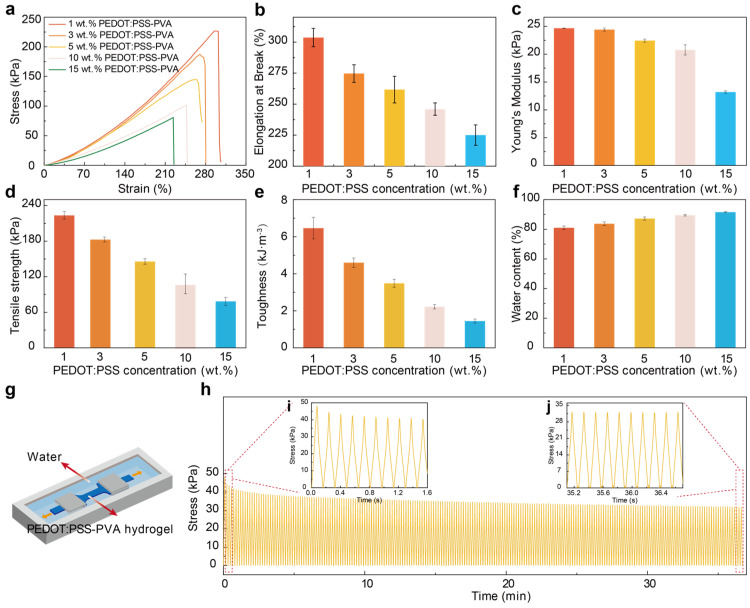
Mechanical performances of the self-healable PEDOT:PSS-PVA nanocomposite hydrogel. (**a**) Tensile stress–strain curves of the PEDOT:PSS-PVA hydrogel with 1 wt. % (red), 3 wt. % (orange), 5 wt. % (yellow), 10 wt. % (pink), and 15 wt. % (blue) PEDOT:PSS contents. Change in elongation at break (**b**) Young’s modulus, (**c**) tensile strength, (**d**) tensile strength, (**e**) toughness, (**f**) water content with different PEDOT:PSS contents. (**g**) Schematic illustration of PEDOT:PSS-PVA hydrogel mechanical properties test. (**h**) Loading–unloading mechanical performance of the 1 wt.% PEDOT:PSS-PVA nanocomposite hydrogels after 200 cycles. Inset: The long-term stability of the 1 wt.% PEDOT:PSS-PVA hydrogel during the first ten cycles (**i**) and last ten cycles (**j**).

Taking advantage of the outstanding mechanical nature of PEDOT:PSS-PVA nanocomposite hydrogels, we comprehensively explore the mechanical performance of free-standing PEDOT:PSS-PVA hydrogels with various PEDOT:PSS contents with 1, 3, 5, 10, and 15 wt.%, and reach ultimate stresses of 226 kPa, 187 kPa, 145 kPa, 101 kPa, and 80.5 kPa and corresponding ultimate strains of 307%, 280%, 274%, 248%, 225%, respectively (Figure 3a). With increasing PEDOT:PSS content, the elongation at break of PEDOT:PSS-PVA nanocomposite hydrogel decreases significantly from 300% to 222%, probably owing to the lower PVA contents leading to less PVA chain entanglement and the existence of dynamic borate ester bonds between borate and PVA chains (Figure 3b). Meanwhile, the Young’s modulus of the PEDOT:PSS-PVA nanocomposite hydrogel maintains a distinct reduction from 24.68 kPa to 13.2 kPa as the PEDOT:PSS content increased from 1 wt.% to 15 wt.% (Figure 3c). With an increment in the content of PEDOT:PSS, more conducting components are incorporated into hydrogels, which is reflected in the decreasing trend of the tensile strength and toughness of PEDOT:PSS-PVA hydrogels (Figure 3d,e). The PEDOT:PSS-PVA hydrogels display widely adjustable tensile strengths ranging from 223 kPa to 78 kPa and a toughness ranging from 6.46 kJ m^−3^ to 1.446 kJ m^−3^. Note here that 3 wt.% PEDOT:PSS-PVA hydrogel displays a favorable self-healing efficiency and mechanical performance with a stress of 186 kPa and strain of 270%. Additionally, the water content of PEDOT:PSS hydrogels gradually improved with the increase in the PEDOT:PSS contents (Figure 3f). This improved water content of PEDOT:PSS-PVA hydrogels can be attributed to the increment of PSS chains that possess hydrophilic properties, which is in good agreement with the descending trend of the Young’s modulus. We further explore the stability of 1 wt.% PEDOT:PSS-PVA hydrogels (Figure 3g) by performing multiple loading–unloading tests after 200 and 400 cycles (Figure 3h–j and Figure S4). The introduction of the PVA network and multiple reversible dynamic interactions provide dynamic double polymer networks and bonds, wherein the structure of the PEDOT:PSS-PVA network dissipated the deformation energy reversibly to guarantee the production of PEDOT:PSS-PVA hydrogels with excellent mechanical stability [24,32,35,36].

The self-healiablity of PEDOT:PSS-PVA nanocomposite hydrogels also plays a role in imparting such hydrogels with favorable mechanical stability. During the process of cyclic loading and unloading, the PEDOT:PSS-PVA hydrogel network may experience fractures that are detrimental to the mechanical stability. Fortunately, the self-healiability of the PEDOT:PSS-PVA hydrogels can rapidly repair the mechanical network, mitigating the effects of fatigue and damage and preventing the further propagation of injuries, thus maintaining the long-term mechanical stability of the PEDOT:PSS-PVA hydrogel.

Additionally, the self-healing property and mechanical performance of PEDOT:PSS-PVA nanocomposite hydrogels with different thickness (1 mm, 1.5 mm, and 3 mm) are explored as shown in Figure S5. As the thickness of PEDOT:PSS-PVA nanocomposite hydrogels increases, the mechanical performance is significantly improved. The dense molecular chain arrangement among PEDOT:PSS chains and PVA chains and the abundant boric ester bonds between PVA and borax endow the PEDOT:PSS-PVA nanocomposite hydrogel with a stable structure, which is the main reason for such hydrogels exhibiting favorable mechanical properties. Benefiting from the reversible dynamic borate ester bonding between borate and PVA chains, PEDOT:PSS-PVA nanocomposite hydrogels with varying thickness exhibit favorable self-healability. The thicker the PEDOT:PSS-PVA hydrogel, the longer it takes to repair, and such hydrogels typically experience reorganization defects. A PEDOT:PSS-PVA hydrogel with a thinner thickness will demonstrate weakened mechanical properties after self-healing. Additionally, due to the incomplete self-healing of PEDOT:PSS-PVA nanocomposite hydrogels, the mechanical properties of such self-healable hydrogels are worse than those for the original. Therefore, we select 1.5 mm PEDOT:PSS-PVA nanocomposite hydrogel, which is able to maintain its mechanical and self-healing performance as a sensing material, to prepare strain sensors, aiming to monitor human motions. These favorable mechanical properties laid a better foundation for the application of this PEDOT:PSS-PVA nanocomposite hydrogel as a sensing material. 

Compared to previously reported PEDOT:PSS-based hydrogels and self-healing materials (Table 1), our PEDOT:PSS-PVA nanocomposite hydrogel exhibits favorable mechanical and self-healing performance. All these attractive outcomes make the PEDOT:PSS-PVA nanocomposite hydrogel one of the optimal candidates for strain sensor applications. Furthermore, the excellent and stable mechanical performance of PEDOT:PSS-PVA hydrogels is particularly desirable for strain sensors, while their performance can be further enhanced by post-treatment methods. All these attractive outcomes make our PEDOT:PSS-PVA nanocomposite hydrogel one of the optimal candidates for strain sensor applications [6,24]. 

#### 3.1.3. Sensing Performance

**Figure 4:**
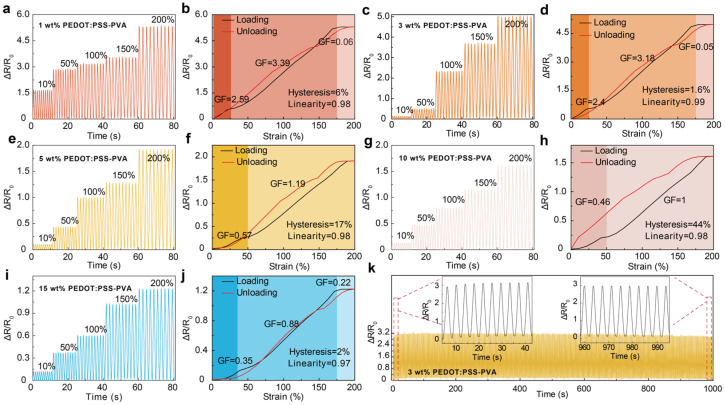
Sensing properties of the PEDOT:PSS-PVA nanocomposite hydrogel strain sensors. Strain sensing performance of the (**a**) 1 wt.%, (**c**) 3 wt.%, (**e**) 5 wt.%, (**g**) 10 wt.%, and (**i**) 15 wt.% PEDOT:PSS-PVA hydrogels under various strains. Relative resistance change of the (**b**) 1 wt.%, (**d**) 3 wt.%, (**f**) 5 wt.%, (**h**) 10 wt.%, and (**j**) 15 wt.% PEDOT:PSS-PVA hydrogel strain sensors under different applied strains. (**k**) Strain sensing performance of the mechanical stability of the 3 wt.% PEDOT:PSS-PVA conducting polymer hydrogel at 200 cycles.

Sensitivity is a crucial element for designing strain sensors, which is beneficial to catch subtle human motions or physiological signals [3,8,30,40]. Therefore, the sensing performance of PEDOT:PSS-PVA nanocomposite hydrogel strain sensors with various PEDOT:PSS contents is systematically investigated. PEDOT:PSS-PVA hydrogels display a broad working range from 10% to 200% (Figure 4a,c,e,g,i), and show stable and reliable sensing performance. Furthermore, by increasing the PEDOT:PSS contents from 1 wt.% to 15 wt.%, the obtained PEDOT:PSS-PVA hydrogel exhibits various GF, in which the 3 wt.% PEDOT:PSS-PVA hydrogel displays high sensitivity—1–25% with a GF of 2.4, 25–175% with a GF of 3.18, and 175–200% with a GF of 0.05 (Figure 4d). The diverse PEDOT:PSS content is the main reason for the variation in relative resistance of PEDOT:PSS-PVA hydrogels. As the solid content of PEDOT:PSS increases, the conductive network gradually becomes denser, leading to less change in the conductive network and a decreasing trend in sensitivity (Figure 4). When PEDOT:PSS-PVA nanocomposite hydrogels are stretched, the internally conducting network of such hydrogels with a higher PEDOT:PSS content becomes more compact, resulting in a diminished relative resistance change of the PEDOT:PSS-PVA hydrogel. Subsequently, as the stress continues to be applied, the conductive network of PEDOT:PSS-PVA hydrogels is gradually disconnected and the conductive pathways become fewer in number. At this point, the sensing mechanism of PEDOT:PSS-PVA hydrogel strain sensors transitions from the break mechanism to a tunneling effect, which is the reason for the sensing materials exhibiting various sensitivities at different strains. Therefore, PEDOT:PSS-PVA nanocomposite hydrogels with excessive and compact nanostructures will display reduced sensitivity under equivalent strain conditions. Hysteresis and linearity are also key parameters for evaluating the sensing performance of strain sensors [41]. Consequently, we further characterize the hysteresis and linearity of PEDOT:PSS-PVA hydrogel sensors with different PEDOT:PSS contents to illustrate their superior sensing properties under various strains (Figure 4b,d,f,h,j). PEDOT:PSS-PVA composite hydrogels all exhibit excellent linearity (0.97–0.99) and display different hysteresis behaviors. Among them, the 3 wt.% PEDOT:PSS-PVA hydrogel strain sensor shows low hysteresis (1.6%) and excellent linearity (0.99). Stability is fundamental for strain sensors due to the fact that it guarantees repeatable and reliable usage in a variety of applications, particularly for rapid or long-term monitoring. The uniaxial cyclical tensile loading test at a strain of 100% is performed to further investigate the sensing stability and the mechanical robustness of PEDOT:PSS-PVA hydrogels. As shown in Figure 4e, the 3 wt.% PEDOT:PSS-PVA hydrogel still yields stable signals after 200 stretching cycles, indicating that this hydrogel possesses remarkable electrical and sensing stability (Figure 4k).

### 3.2. Applications as Wearable Electronic Skins for Human Motion Detection

**Figure 5:**
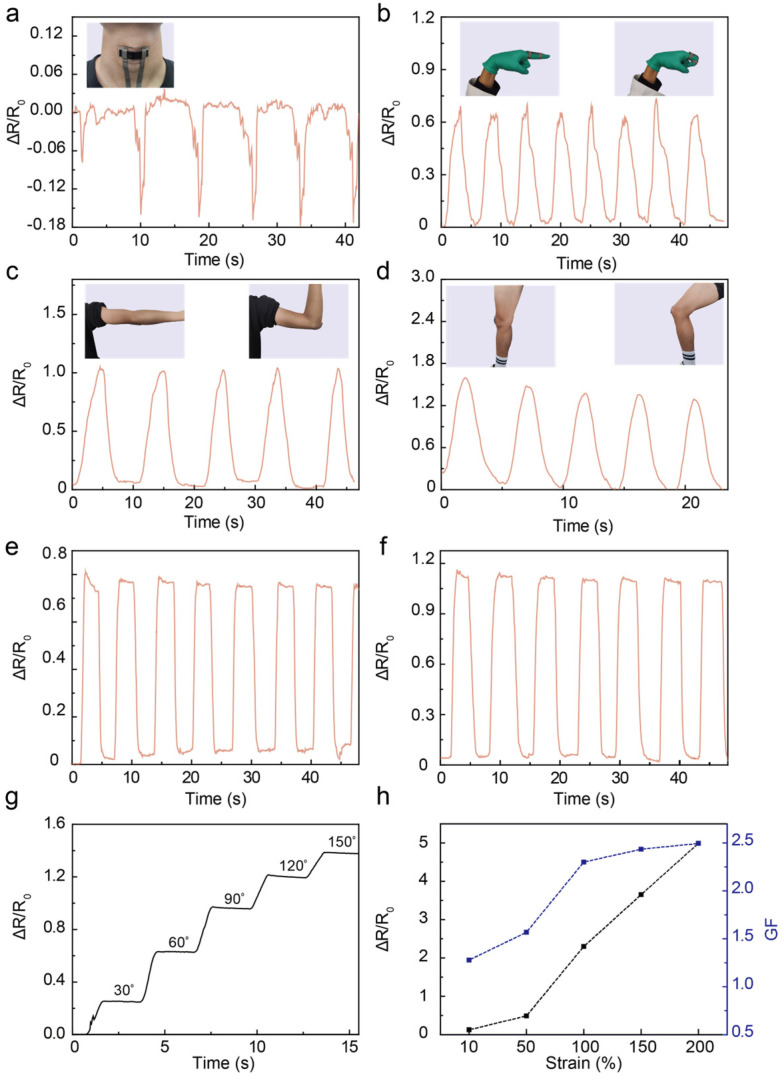
Self-healable PEDOT:PSS-PVA nanocomposite hydrogel strain sensors for detecting human motions. PEDOT:PSS-PVA hydrogel strain sensor is used to monitor swallowing (**a**), finger bending motion (**b**), elbow flexion motion (**c**), knee flexion motion (**d**), finger bending motion (**e**), and knee flexion motion (**f**) after PEDOT:PSS-PVA hydrogel self-healing. (**g**) Monitoring different angles in real-time using the PEDOT:PSS-PVA hydrogel strain sensor. (**h**) Relative changes in resistance and GF of the PEDOT:PSS-PVA nanocomposite hydrogel strain sensors under varying strains.

Taking advantage of PEDOT:PSS-PVA nanocomposite hydrogel’s high sensitivity under board deformation and its facile fabrication and integration, PEDOT:PSS-PVA hydrogel strain sensor-based wearable electronic skins can offer an attractive route for detecting a variety of physiological signals. To quantify the effectiveness of PEDOT:PSS-PVA hydrogel stain sensors in monitoring human motions, we fabricated a 3 wt.% PEDOT:PSS-PVA hydrogel strain sensor and collected a series of physiological signals such as knee bending, finger bending, and swallowing (Figure 5). The as-prepared PEDOT:PSS-PVA hydrogel strain sensor can recognize subtle swallowing motions and still maintain a favorable attachment to the throat (Figure 5a). Additionally, the PEDOT:PSS-PVA hydrogel strain sensor not only shows excellent responses to subtle physiological signals but also to larger-scale human activities. Owing to the broad working range of PEDOT:PSS-PVA hydrogel strain sensors, we further investigate the stability and repeatability of the relative resistance of PEDOT:PSS-PVA hydrogels under large deformations, such as finger bending (Figure 5b) and joint flexion of the arm and knee (Figure 5c,d). For example, the knee-attached sensor can detect large deformation, and the corresponding electrical signal changes significantly, which indicates the high sensitivity of PEDOT:PSS-PVA hydrogel strain sensors (Figure 5d). We further conduct the resistance vibration of healed PEDOT:PSS-PVA hydrogel strain sensors under large deformation like finger and knee bending (Figure 5e,f), and more bending angles (Figure 5g). During the bending process of PEDOT:PSS-PVA hydrogel strain sensor, the relative resistance of such strain sensors continuously rise with the increment in the bending angle. The prepared PEDOT:PSS-PVA hydrogel strain sensor still displays a rapid response and high sensitivity under various large strains. To illustrate the stability of PEDOT:PSS-PVA hydrogel strain sensors, we examine the weight and sensing performance change of the prepared strain sensor (Figure S6). Benefitting from the stabilized properties of PEDOT:PSS-PVA hydrogels and the excellent sealing performance of 3M VHB, the obtained strain sensor (3 cm × 2 cm) shows stable weight without obvious water loss change and reliable sensing performance after 9 days. Further results demonstrate that the relative resistance change of PEDOT:PSS-PVA hydrogel strain sensors linearly increases with the applied strain. Additionally, the GF of such strain sensors can also be calibrated with the supplied strain (Figure 5h). The prepared self-healed PEDOT:PSS-PVA hydrogel strain sensor still retains favorable accuracy regarding sensing signals and the stability of sensitivity, which demonstrates its promising future in various fields including electronic skins, soft robotics, and medical detection.

## 4. Conclusions

In this study, a self-healable PEDOT:PSS-PVA nanocomposite hydrogel is designed via the physical–chemical dual crosslinking of PEDOT:PSS nanofibers and PVA. With multiple reversible dynamic bonds that consist of dynamic boronic-ester covalent bonds, hydrogen bonds, and electrostatic interactions, the resultant PEDOT:PSS-PVA nanocomposite hydrogel exhibits superior sensing performance and self-healability. Enabled by this capability, we further fabricate hydrogel strain sensor-based wearable electronic skins to monitor various physiological signals, which display repeatable and stable sensing performance. The current study not only addresses a lingering problem in the irreversible mechanical damage of hydrogels under significant deformation, but also provides an attractive novel avenue with regard to strain sensors.

## Figures and Tables

**Table 1:** Comparison of mechanical and self-healing properties of the PEDOT:PSS-PVA nanocomposite hydrogel with previously reported PEDOT:PSS-analogous hydrogels and some self-healing conductive hydrogels.

Hydrogel Sample	Preparation Method	Strain (%)	Self-Healing Efficiency (%)	Refs.
PEDOT:PSS-PVA	Physical crosslinking	300	---	[6]
PEDOT:PSS-PVA	Physical crosslinking	519.9	---	[37]
PEDOT:PSS-PVA	Physical crosslinking	600	---	[24]
PEDOT:PSS-PVA	Physical–chemical dual crosslinking	300	---	[29]
PEO terminated with dithiodipropionic	Chemical crosslinking	650	50	[38]
PANI/PVA/ CPBA/Ca^2+^	Chemical crosslinking	633	6	[39]
PEDOT:PSS-PVA	Physical–chemical dual crosslinking	300	83.5	This work

## Data Availability

The data presented in this study are available on request from the corresponding author. The data are not publicly available due to privacy.

## Supplementary Materials

The following supporting information can be downloaded at: https://www.mdpi.com/article/10.3390/nano13172465/s1. Figure S1. FTIR spectra of PVA, PEDOT:PSS, and PEDOT:PSS-PVA nanocomposite hydrogel. Figure S2. Photographs of the self-healing behavior of physical-crosslinked pure PEDOT:PSS hydrogel (a), PEDOT:PSS-PVA composite hydrogel (b), and physical-chemical crosslinked PEDOT:PSS-PVA nanocomposite hydrogel in this work (c). Figure S3. Resistance recovery of the PEDOT:PSS-PVA nanocomposite hydrogel during the cutting-healing cycle. Figure S4. Loading-unloading mechanical performance of 1 wt.% PEDOT:PSS-PVA nanocomposite hydrogels at 400 cycles. Figure S5. Typical stress-strain curves of the original and healed PEDOT:PSS-PVA nanocomposite hydrogels with (a) 1 mm, (b) 1.5 mm, and (c) 3 mm. Figure S6. (a) Weight changes of PEDOT:PSS-PVA nanocomposite hydrogel strain sensors within 9 days. (b) Strain sensing performance of 3 wt.% PEDOT:PSS-PVA nanocomposite hydrogel strain sensor under various strains after 9 days. Table S1. Compositions of PEDOT:PSS-PVA nanocomposite hydrogels.
