# Supplementary material for: Self-Healable PEDOT:PSS-PVA Nanocomposite Hydrogel Strain Sensor for Human Motion Monitoring
Source: Nanomaterials (Basel). 2023 Aug 31;13(17):2465. doi: 10.3390/nano13172465 (PMC10489763; doi:10.3390/nano13172465)
Supplement: Supplementary file 1 [file nanomaterials-13-02465-s001.zip › nanomaterials-2535614-supplementary.pdf]

SUPPLEMENTARY INFORMATION FOR

Self-Healable PEDOT:PSS-PVA Nanocomposite Hydrogel Strain Sensor for Human Motion Monitoring

Jie Cao[1,2]†, Zhilin Zhang[1]†, Kaiyun Li[1,2], Cha Ma[3], Weiqiang Zhou[1], Tao Lin[4]*, Jingkun Xu[1,5]*, Ximei Liu[1]*

[1] Jiangxi Key Laboratory of Flexible Electronics, Flexible Electronics Innovation Institute, Jiangxi Science and Technology Normal University, Nanchang 330013, China; caojie9905@163.com (J.C.); zhangzhilin1117@163.com (Z.Z.); guchengbaiyi2020@163.com (K.L.); zhouwqh@163.com (W.Z.)

[2] School of Chemistry and Chemical Engineering, Jiangxi Science and Technology Normal University, Nanchang 330013, China

[3] Key Laboratory of Organic Chemistry in Jiangxi Province, Institute of Organic Chemistry, Jiangxi Science and Technology Normal University, Nanchang 330013, China; macha001@126.com (C. M.)

[4] Department of Orthopedics, Qilu Hospital, Cheeloo College of Medicine, Shandong University, Qingdao 266035, China

[5] School of Chemistry and Molecular Engineering, Qingdao University of Science and Technology, Qingdao 266042, China

* Correspondence: osteojoint@sina.com (T.L.); xujingkun1971@yeah.net (J.X.); liuxm@jxstnu.edu.cn (X.L.)

† These authors contributed equally to this work.

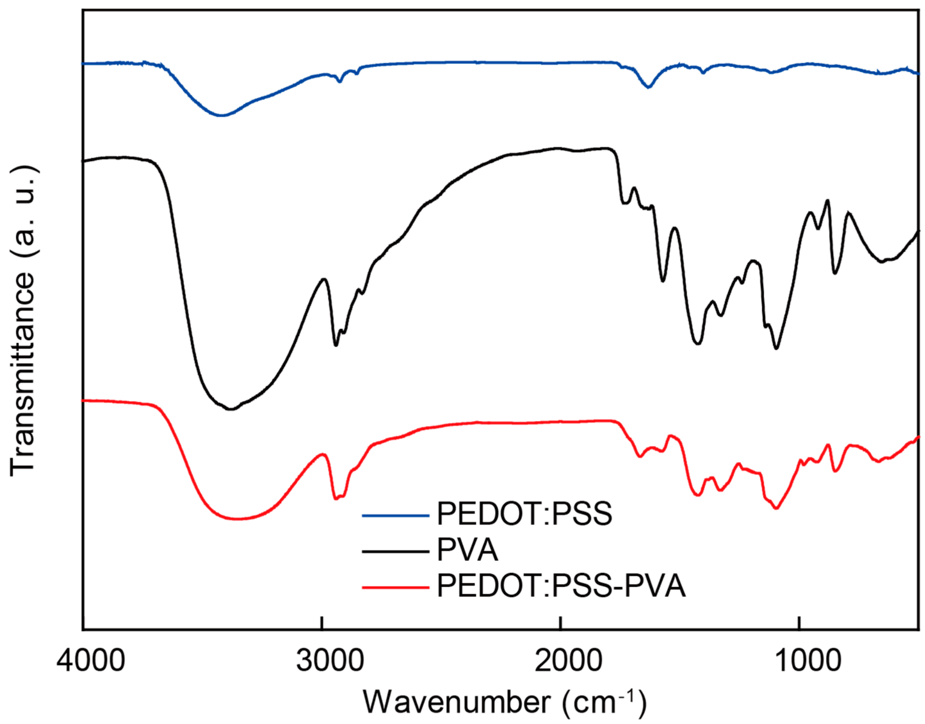

Figure S1. FTIR spectra of PVA, PEDOT:PSS, and PEDOT:PSS-PVA nanocomposite hydrogel.

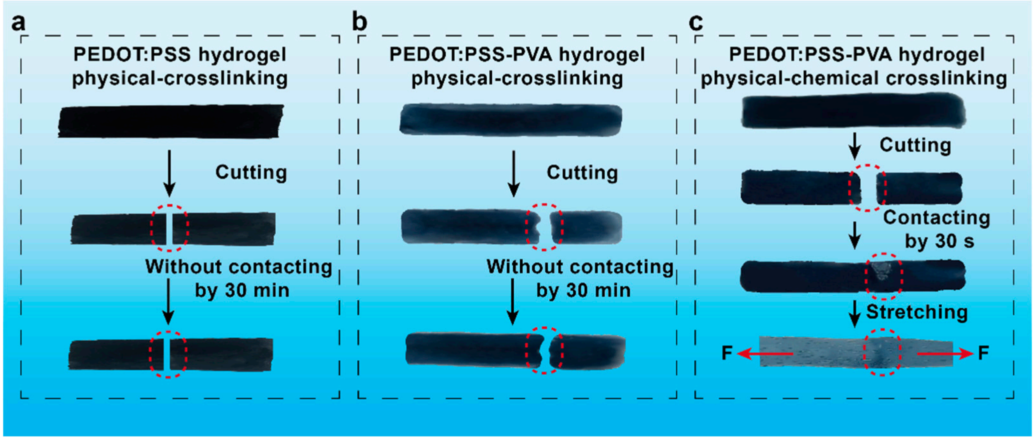

Figure S2. Photographs of the self-healing behavior of physical-crosslinked pure PEDOT:PSS hydrogel (a), PEDOT:PSS-PVA composite hydrogel (b), and physical-chemical crosslinked PEDOT:PSS-PVA nanocomposite hydrogel in this work (c).

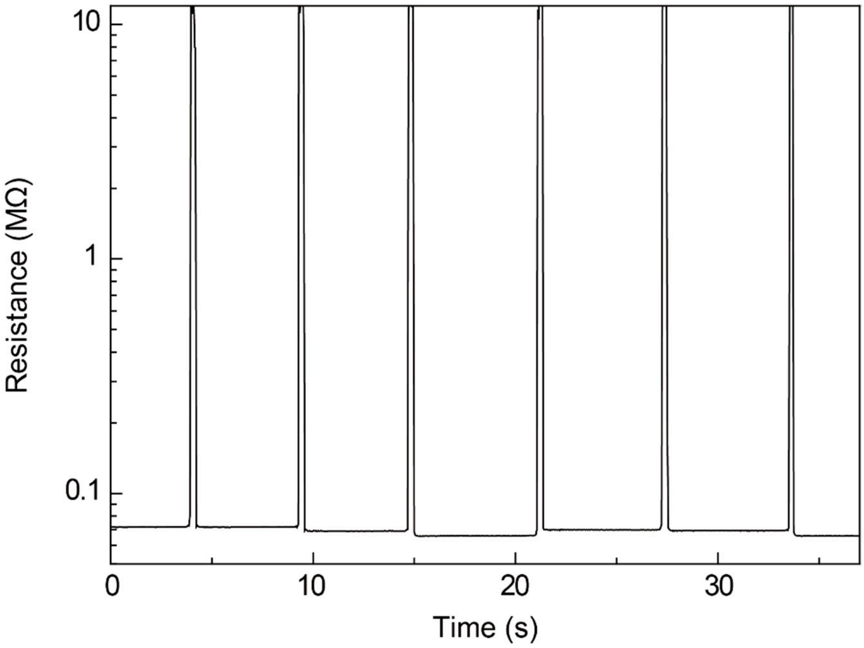

Figure S3. Resistance recovery of the PEDOT:PSS-PVA nanocomposite hydrogel during the cutting-healing cycle.

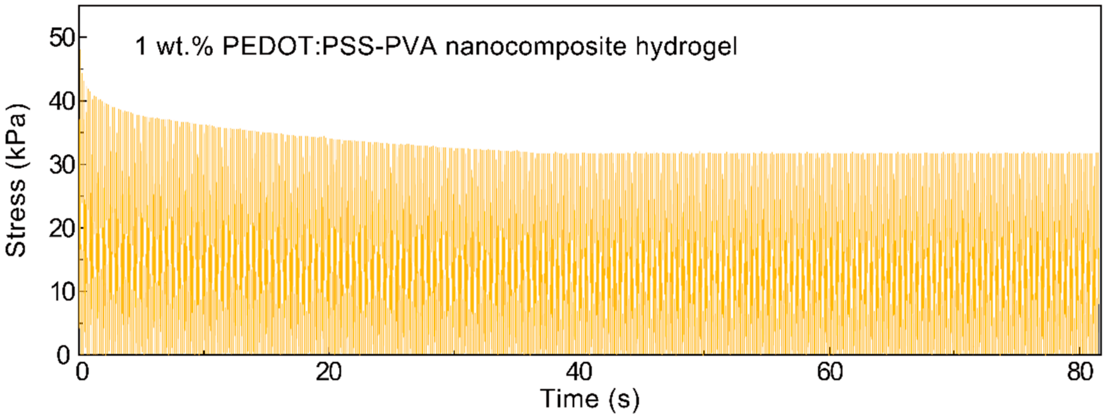

Figure S4. Loading-unloading mechanical performance of 1 wt.% PEDOT:PSS-PVA nanocomposite hydrogels at 400 cycles.

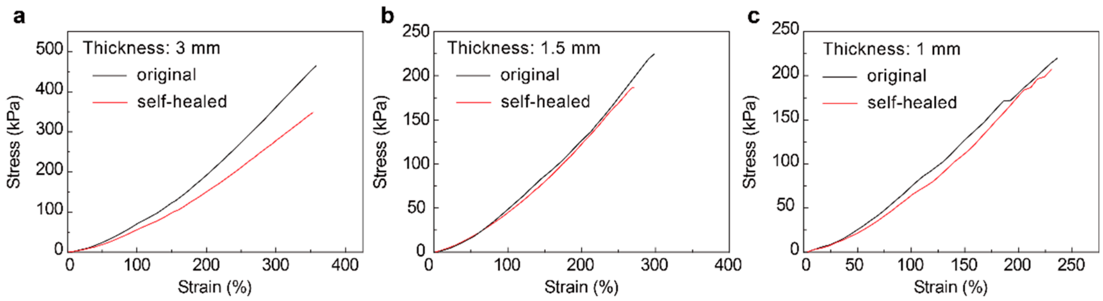

Figure S5. Typical stress-strain curves of the original and healed PEDOT:PSS-PVA nanocomposite hydrogels with (a) 1 mm, (b) 1.5 mm, and (c) 3mm.

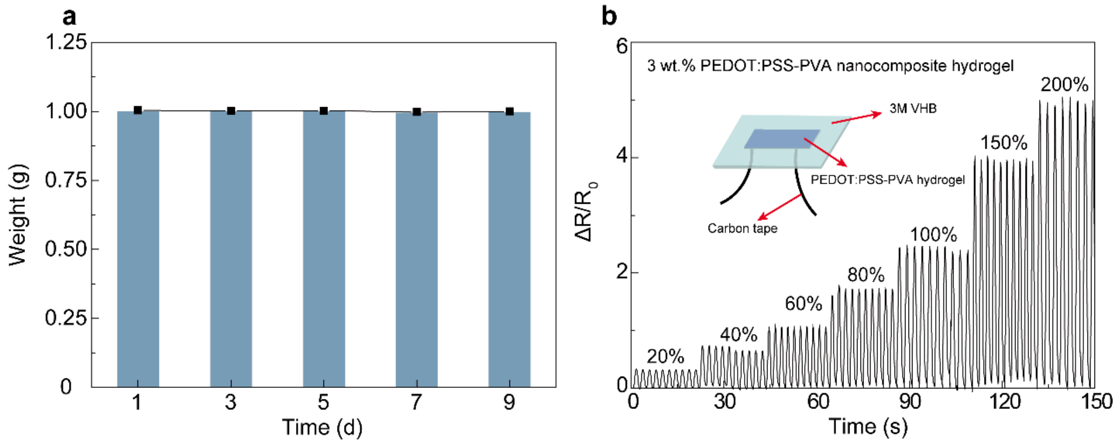

Figure S6. (a) Weight changes of PEDOT:PSS-PVA nanocomposite hydrogel strain sensors within 9 days. (b) Strain sensing performance of 3 wt.% PEDOT:PSS-PVA nanocomposite hydrogel strain sensor under various strains after 9 days.

Table R2 (as Table S1). Compositions of PEDOT:PSS-PVA nanocomposite hydrogels

Hydrogel sample	m (7wt.% PEDOT:PSS)	m (7wt.% PVA)
1 wt.% PEDOT:PSS-PVA	1 g	99 g
3 wt.% PEDOT:PSS-PVA	1 g	32.33 g
5 wt.% PEDOT:PSS-PVA	1 g	19 g
10 wt.% PEDOT:PSS-PVA	1 g	9 g
15 wt.% PEDOT:PSS-PVA	1 g	5.66 g
